# Treatment With Multi-Species Probiotics Changes the Functions, Not the Composition of Gut Microbiota in Postmenopausal Women With Obesity: A Randomized, Double-Blind, Placebo-Controlled Study

**DOI:** 10.3389/fcimb.2022.815798

**Published:** 2022-03-11

**Authors:** Mariusz Kaczmarczyk, Monika Szulińska, Igor Łoniewski, Matylda Kręgielska-Narożna, Karolina Skonieczna-Żydecka, Tomasz Kosciolek, Valentyn Bezshapkin, Paweł Bogdański

**Affiliations:** ^1^ Department of Clinical and Molecular Biochemistry, Pomeranian Medical University in Szczecin, Szczecin, Poland; ^2^ Department of Treatment of Obesity, Metabolic Disorders and Clinical Dietetics, University of Medical Sciences in Poznań, Poznań, Poland; ^3^ Department of Biochemical Sciences, Pomeranian Medical University in Szczecin, Szczecin, Poland; ^4^ Department of Human Nutrition and Metabolomics, Pomeranian Medical University in Szczecin, Szczecin, Poland; ^5^ Małopolska Centre of Biotechnology, Jagiellonian University, Kraków, Poland

**Keywords:** probiotics, microbiota, metabolism, obesity, menopause

## Abstract

Probiotics are known to regulate host metabolism. In randomized controlled trial we aimed to assess whether interventions with probiotic containing following strains: *Bifidobacterium bifidum* W23, *Bifidobacterium lactis* W51, *Bifidobacterium lactis* W52, *Lactobacillus acidophilus* W37, *Levilactobacillus brevis* W63, *Lacticaseibacillus casei* W56, *Ligilactobacillus salivarius* W24, *Lactococcus lactis* W19, and *Lactococcus lactis* W58 affect gut microbiota to promote metabolic effects. By 16S rRNA sequencing we analyzed the fecal microbiota of 56 obese, postmenopausal women randomized into three groups: (1) probiotic dose 2.5 × 10^9^ CFU/day (n = 18), (2) 1 × 10^10^ CFU/day (n = 18), or (3) placebo (n = 20). In the set of linear mixed-effects models, the interaction between pre- or post-treatment bacterial abundance and time on cardiometabolic parameters was significantly (FDR-adjusted) modified by type of intervention (26 and 19 three-way interactions for the pre-treatment and post-treatment abundance, respectively), indicating the modification of the bio-physiological role of microbiota by probiotics. For example, the unfavorable effects of *Erysipelotrichi*, *Erysipelotrichales*, and *Erysipelotrichaceae* on BMI might be reversed, but the beneficial effect of *Betaproteobacteria* on BMI was diminished by probiotic treatment. Proinflammatory effect of Bacteroidaceae was alleviated by probiotic administration. However, probiotics did not affect the microbiota composition, and none of the baseline microbiota-related features could predict therapeutic response as defined by cluster analysis. Conclusions: Probiotic intervention alters the influence of microbiota on biochemical, physiological and immunological parameters, but it does not affect diversity and taxonomic composition. Baseline microbiota is not a predictor of therapeutic response to a multispecies probiotic. Further multi-omic and mechanistic studies performed on the bigger cohort of patients are needed to elucidate the cardiometabolic effect of investigated probiotics in postmenopausal obesity.

## Introduction

Cardiometabolic risk factors (CMRFs), including obesity, abnormal lipid profile hypertension, insulin resistance, and aberrant glycemic control, play a role in the pathogenesis of cardiovascular diseases (CVD), which is one of the leading causes of mortality. Some important causes of this disease are the consumption of high-calorie foods combined with a sedentary lifestyle. Due to the high prevalence of CVD, effective and safe methods to reduce CMRFs are being sought. One such intervention includes probiotic administration, which may have beneficial effects on some CMRFs (Skonieczna-Żydecka et al., 2020). Probiotics are live microorganisms that can confer health benefits to the host when administered in adequate amounts (Hill et al., 2014). Despite the encouraging clinical effects associated with probiotic intake, their mechanism of action is often unclear, and mechanistic analyses are necessary to assess the efficacy (Kristensen et al., 2016). A study by Szulińska et al. (2018b) showed favorable effects of the administration of multistrain probiotics (PB) on glucose metabolism, lipid profile, waist circumference, visceral fat, serum uric acid level, and lipopolysaccharide (LPS) concentration in obese, postmenopausal women. Moreover, it was also revealed that this bacterial consortium improved both functional and biochemical markers of vascular function and reduced homocysteine concentration, oxidative stress, and inflammation. It was hypothesized that the above-mentioned formula might improve epithelial barrier integrity, serving as an inhibitor of pro-inflammatory cytokine synthesis and as an effective tool for decreasing the endotoxin load (Hemert and Ormel, 2014; Szulińska et al., 2018b; Sabico et al., 2019). However, the mechanism of action of this formula is still poorly understood. In a recent published study authors have identified markers of cardiovascular diseases and metabolic syndrome based on gut microbiota changes, which can be used for development of targeted microbiota correction in the treatment and prevention of these diseases (Meleshko et al., 2021). One hypothesis explaining mode of action of the investigated bacterial consortium is the modification of the gut microbiota composition, which counteracts the adverse changes associated with CVD risk. To date, the microbiota after PB administration has not been analyzed in individuals suffering from metabolic disorders, and the results observed after the application of this bacterial consortium in other groups of patients are inconclusive (Chahwan et al., 2019; Horvath et al., 2020a).

To determine the potential role of microbiota modification in cardiovascular risk reduction, we analyzed the microbiota in a group of obese postmenopausal women receiving PB in a randomized clinical trial (RCT). The study aimed to verify the following research hypotheses: 1) the administration of PB causes changes in the microbiota, which may be responsible for the beneficial metabolic effects; 2) changes in biochemical and physiological parameters during the study correlate with baseline and end-study microbiota; 3) baseline gut microbiota is different between therapy responders and non-responders.

## Materials and Methods

This study analyzed the fecal microbiota in a population of obese postmenopausal women who underwent a 12-week, single-center, randomized, double-blind, placebo-controlled clinical trial in which PB was administered. It was conducted at the Department of Education and Treatment of Obesity and Metabolic Disorders University of Medical Sciences in Poznań, Poland. The protocol was registered at the U.S. National Institute of Health (ClinicalTrials.gov; Identifier: NCT03100162). Ethical approval was obtained from the Bioethical Committee of Poznan University of Medical Sciences (No. 871/2015) and prior written informed consent was obtained from all participants. The informed consent allowed samples to be used for future analyses. The study took place from 27 February 2016 to 31 December 2017. The material obtained during this study was analyzed in a multidirectional manner, and the results were presented in peer-reviewed scientific publications (Szulińska et al., 2018a; Szulińska et al., 2018b; Majewska et al., 2020).

### Subjects

The study population has been described in detail previously (Szulińska et al., 2018b). A total of 110 postmenopausal obese women were invited to participate in the study. The inclusion criteria were as follows: (1) women aged 45–70 years, (2) ≥1 year since last menstruation, (3) body mass index (BMI) 30–45 kg/m^2^, (4) abdominal obesity-related waist circumference > 80 cm (International Diabetes Federation 2005); (5) body fat content, assessed by electrical bioimpedance at ≥33%; and (6) stable body weight in the month before the trial (permissible deviation ±1 kg). The following criteria excluded participants from the study: (1) secondary form of obesity; (2) gastrointestinal diseases; (3) diabetes; (4) pharmacotherapy for hypertension or dyslipidemia in the three months before the trial; (5) history of use of any dietary supplements in the three months before the study; (6) intake of antibiotics within one month before the study; (7) clinically significant acute inflammation; (8) nicotine, alcohol, or drug abuse; (9) participation in weight management studies or use of medications known to alter food intake or bodyweight; (10) vegetarian dietary habits; (11) use of prebiotics- and probiotics-enriched products (for at least three weeks before the screening) and products with a high content of dietary fibre or intake of high quantities of fermented food (> 400 g/day); (12) hormone replacement therapy. Compliance with any of the above exclusion criteria during the trial resulted in the immediate cessation of participation in the study. Based on the inclusion and exclusion criteria, 29 women did not qualify for the study, and 81 women diagnosed with obesity were eligible. They were randomly assigned to the placebo or probiotic group, and this distribution was unknown to both, the principal investigators and the participants. Finally, 71 participants in the placebo group (PL, n = 24), low probiotic dose group (LPD, n = 24), and high probiotic dose group (HPD, n = 23) completed the 12-week intervention. Stool samples were collected into sterile, plastic containers and stored until analyses at -80C. Microbiome analysis was carried out in 56 women (20 in the PL group, 18 in the LPD group, and 18 in the HPD group) for whom the next-generation sequencing of stool samples yielded at least 10,000 reads. A flowchart of this study is shown in **Supplementary Figure 1**.

### Probiotic Supplements and Allocation

All eligible and consenting participants were assigned unique codes as identifiers. They were allocated (1:1:1) to receive either the probiotics (higher - HPD or lower dose - LPD) or a placebo (PL). The randomization was computer-generated using permuted blocks with a block size of four (Winclove AB, Amsterdam, The Netherlands). The research personnel involved in the study were not able to adjust the randomization or discern which product the participants were receiving, ensuring allocation concealment. The probiotic group received sachets containing 2 g of freeze-dried powder of the probiotic mixture from Ecologic^®^ Barrier (Winclove). The HPD group received 1 × 10^10^ colony forming units (CFU) per day divided into two equal doses, whereas the LPD group received 2.5 × 10^9^ colony forming units (CFU) per day divided into two equal doses. The PB contained nine bacterial strains: *Bifidobacterium bifidum* W23, *Bifidobacterium lactis* W51, *Bifidobacterium lactis* W52, *Lactobacillus acidophilus* W37, *Levilactobacillus brevis* W63, *Lacticaseibacillus casei* W56, *Ligilactobacillus salivarius* W24, *Lactococcus lactis* W19, and *Lactococcus lactis* W58. All strains were present in approximately equal amounts, and the quality of the study batch was tested every three months to confirm the viability of the strains. The placebo group received the same sachets containing only the excipients maize starch and maltodextrins. The placebo was indistinguishable in color, smell, and taste from the probiotic formulation. The contents were dissolved in a glass of water at room-temperature and all participants consumed two sachets per day, one before breakfast and one before going to bed. They were asked to return every four weeks to hand back the empty sachets and were given fresh refills to monitor their compliance with the study protocol. They were also asked to not change their routine physical activity and diets and report any side effects.

### Anthropometric and Biochemical Measurement

At enrollment and after 12 weeks of treatment, anthropometric parameters were evaluated, and laboratory tests were performed for each group. All measurements were recorded after an overnight fast. The methods are described previously (Szulińska et al., 2018a; Szulińska et al., 2018b) and included the following parameters: 1/anthropometric: weight (weight scale, metric stadiometer), waist circumference (tape measure), body composition (Bioscan 920-2); 2/vascular: blood pressure (sphygmomanometer - Omron Healthcare), pulse wave velocity and analysis (sphygmomanometer - Sphygmocor Px), augmentation index, aortic pressure and pulse pressure (applanation tonometry); 3/biochemical: glucose, uric acid, lipid profile (Lm Integrated Chemistry System Analyzer), Insulin (Immunoradiometry - Diasource Immunoassays S.A.), Lipopolysaccharide (Lps) (Kinetic Assay -Lonza, Walkersville), Tumor Necrosis Factor (TNF) -A (Enzyme Immunoassay - DRG Instruments Gmbh), Interleukin (Il) -6 (Elisa - Drg Instruments Gmbh), vascular endothelial growth factor (vegf) (Elisa - R&D Systems), Thrombomodulin (ELISA/American Diagnostica Inc., Stamford), Von Willebrand Factor (ELISA/R&D Systems, Minneapolis).

### 16s rRNA Sequencing

For amplicon analysis, PCR primers 27F (5’ - AGA GTT TGA TCC TGG CTC AG) and 338R (3’ - T GCT GCC TCC CGT AGG AGT) were used to target the V1-V2 region of the 16S rRNA gene. The 2 × 300 bp paired-end sequencing was performed on an Illumina MiSeq platform. An initial quality check was performed using FastQC, and the 16S sequences (sequence count summary: minimum 16,620, median 23,549.5, maximum 53,363) were processed using QIIME 2 (Bolyen et al., 2019). Paired-end reads were joined using the q2-vsearch (Rognes et al., 2016) plugin (sequence count summary: minimum 9,248, median 13,331.5, maximum 41,011). Quality filtering of joined reads was performed using the quality-filter plugin with a minimum quality score of 30. Next, joined and quality-filtered sequences (sequence count summary: minimum 9,246, median 13,328.5, maximum 41,004) were subjected to a denoising strategy using Deblur (Amir et al., 2017) with the following parameters (left trimming 35 and length trimming 300) which allowed to resolve sequence data into 4,716 single sequence variants (sub operational taxonomic units, sOTUs) with the following frequency (count) per sample (n = 112) summary: minimum 3,777, median 6,689, maximum 25,058. Taxonomic assignment was performed using a naive Bayes classifier (trained with the reference sequences trimmed to the region V1-V2) against the Greengenes reference database (version 13.8). For better species-level classification accuracy, instead of uniform species distribution, species-dependent prior probabilities (taxonomic weights) assembled with the q2-clawback plugin (Kaehler et al., 2019) were used. Predicted functional profiles were created using PICRUSt2 plugin for QIIME2 (Douglas et al., 2019). Hidden state prediction was performed using maximum parsimony method. The Nearest Sequence Taxonomy Index (NSTI) which controls the distance between query sOTUs and reference phylogeny was set to 2. Predicted MetaCyc pathway abundances inferred by PICRUSt2 were taken for further analysis.

### Machine Learning

For machine learning model development, the relative abundance of microbial features was used as an input. Relative counts were used with a threshold of 0.1% to filter out the rare OTUs. Due to a non-normal, right-skewed distribution (most bacterial relative frequencies were under 1%), a Yeo-Johnson transformation was applied to the dataset. The random forest algorithm was implemented using the scikit-learn library (Pedregosa et al., 2011) in Python. Recursive feature selection and random search optimization of hyperparameters were implemented for model tuning. A 3-fold cross-validation was used to prevent overfitting. ROC and PRC plots were used for the model diagnostics. Feature importance was extracted from trained models and examined using SHAP values (Lundberg et al., 2020).

### Statistical Analysis

Normality in the distribution of participants characteristics was checked using the Shapiro-Wilk test. Baseline characteristics were compared between groups (PL, LPD, HPD; responders, non-responders) using either ANOVA or Kruskal-Wallis test. Alpha diversity was assessed using four metrics (number of sOTUs observed in the sample, Pielou’s evenness, Shannon’s diversity, and Faith’s PD) from the rarefied samples down to 3,777 sOTUs). General linear mixed-effects models were used to compare the metrics between the time points and interventions. The significance of the effects was tested using a likelihood ratio test implemented in the lmtest R package, followed by an adjustment for false discovery rate (FDR) using the Benjamini-Hochberg method among alpha-diversity metrics. The Bray-Curtis distance was used to calculate a dissimilarity matrix of the rarefied sOTUs (down to a minimum count of 3,777) among samples for univariate analysis as follows: Wilcoxon rank-sum test – (1) for the overall intersample distance between time points and (2) for the intersample (baseline) vs. intrasample (same sample) distance difference; general linear mixed-effects models followed by *post-hoc* Wilcoxon rank-sum test between time points for the intervention-specific intersample distances between time points; Kruskal-Wallis test for the intrasample (same sample) distance between interventions. Multivariate permutational analysis of variance (PERMANOVA) was performed to compare the microbial composition between the intervention and time. PERMANOVA tests were computed using the adonis function in the vegan package (2.5-7). To take into account the repeated measures aspect of the design, a subject ID was included as ‘strata’ in the adonis function.

To investigate whether gut microbiota can modulate treatment-related changes in clinical and biochemical features during the study, two sets of general linear mixed-effects models were fitted. Before the analysis, outliers were removed from the vWF and PWA PP, and CRP, HDL, and TG were log-transformed. The first set included the baseline (pre-treatment) taxonomic abundance) and the interaction between time and intervention (group). The effect of the baseline abundance was tested by comparing two nested models with the time by group interaction and the three-way interaction of time by group (intervention) by pre-treatment abundance using a likelihood ratio test. The second set of models included the post-treatment taxonomic abundance and the interaction between time and intervention (group). The effect of the change in abundance was then tested by comparing two nested models (with the time by group interaction and the three-way interaction of time by group by post-treatment abundance after controlling for pre-treatment abundance) using a likelihood ratio test. In both sets of models, the abundance was treated as a time-invariant covariate and was grand-mean centered.

In all models, to account for the compositionality of abundance count data, 128 Monte Carlo instances were generated for each sample, and each instance was then converted using the centered log-ratio transformation using the ALDEx2 package. Models were fitted for each instance, and the results were averaged over 128 instances (P values and coefficients).

A two-stage approach was used to identify the responders and non-responders. First, post- and pre-treatment differences were calculated for each parameter which was then standardized by the mean standard deviation according to the following formula:


$$s=\sqrt{\frac{s_{1}^{2}+s_{2}^{2}}{2}}$$

In the second step, partition around medoids (PAM) clustering technique with Manhattan distance with *a priori* number k = 2 was used to identify possibly least overlapping groups that would correspond to responders and non-responders. The following sets of variables were considered: systolic and diastolic blood pressure (blood pressure response; CRP, TNF, IL-6, and LPS [inflammatory response]; BMI, glucose, insulin [metabolic response]; BMI, TBW, subcutaneous fat, visceral fat, fat mass, free fatty mass (body composition response); total cholesterol, HDL, LDL, TG (lipid response); PWA LAX, PWA AP, PWA PP, PWA SP, PWV, and VEGF (vascular response). The analysis was continued if sufficient evidence of structure in the data was found, as defined by the average silhouette value greater than 0.30.

### Power Analysis

For the mixed effect models, the powerlmm R package (power analysis for longitudinal multilevel/linear mixed-effects models with missing data and unbalanced designs) was used. Here, we assumed a standardized effect size (the post-test difference) of 0.2 and 0.5, the intraclass correlation of 0.5, random intercept model, and probiotic groups were combined). The small effect (0.2 Cohen’s post-test differences) could be detected with a power of 11% and medium effects (0.5 of Cohen’s post-test differences) with a power of 42%.

## Results

### Demographic, Clinical Characteristics, Metabolic Parameters, and Baseline Comparisons Between Groups

A total of 56 women were, at random, given a lower probiotic dose (LPD, n = 18), a higher probiotic dose (HPD, n = 18), or a placebo (PL, n = 20). No serious adverse reactions in the participating postmenopausal women with obesity were reported following the consumption of the multispecies probiotic supplement throughout the study. They did not require any additional medical treatment during the study. The characteristics of the women are shown in **Table 1**. Participants had a mean (SD) age of 56.2 years (7.3) and mean (SD, range) BMI of 35.9 kg/m^2^ (4.0, 29.9–47.5). Baseline characteristics (demographic, clinical, body composition, and metabolic) did not differ between the groups (Q > 0.05). The results of biochemical and vascular analyses have been described previously (Szulińska et al., 2018b; Szulińska et al., 2018a).

**Table 1:** Baseline characteristics per intervention group.

	Placebo(n = 20)	LPD(n = 18)	HPD(n = 18)	Q
Age (years)	58 ± 8	56 ± 7	54 ± 7	0.757
Body mass (kg)	93 ± 12	94 ± 11	93 ± 13	0.976
BMI (kg/m^2^)	36 ± 4	36 ± 4	36 ± 5	0.954
Subcutaneous fat area (cm^2^)	292 ± 59	280 ± 76	290 ± 52	0.954
Visceral fat area (cm^2^)	229 ± 67	221 ± 58	205 ± 45	0.800
Waist circumference (cm)	110 ± 8	111 ± 9	108 ± 9	0.911
Fat mass (kg)	48 ± 11	48 ± 9	47 ± 10	0.976
Fat %	52 ± 8	52 ± 5	50 ± 6	0.806
Fat free mass (kg)	43 ± 8	45 ± 5	46 ± 5	0.800
Fat free %	45 ± 9	47 ± 5	48 ± 8	0.800
TBW (liters)	33 ± 6	34 ± 4	35 ± 5	0.806
HR (bpm)	72 ± 5	75 ± 9	79 ± 9	0.247
SBP (mmHg)	132 ± 13	137 ± 8	132 ± 11	0.800
DBP (mmHg)	83 ± 8	83 ± 6	80 ± 9	0.830
PWA Alx	32 ± 12	33 ± 11	33 ± 12	0.976
PWA AP (mmHg)	15 ± 10	14 ± 6	13 ± 7	0.954
PWA PP (mmHg)	42 ± 11	44 ± 7	43 ± 8	0.954
PWA SP (mmHg)	125 ± 12	130 ± 13	131 ± 8	0.757
PWV (m/s)	7.1 ± 1.2	7.0 ± 0.8	7.4 ± 0.9	0.800
Total cholesterol (mg/dL)	205 ± 35	219 ± 46	220 ± 37	0.830
LDL-C (mg/dL)	120 ± 36	131 ± 49	125 ± 34	0.954
HDL-C (mg/dL)	54 (15)	58 (12)	54 (19)	0.800†
TG (mg/dL)	144 (91)	120 (59)	160 (35)	0.757†
Uric acid (mmol/L)	5.4 ± 1.3	5.4 ± 0.8	6.0 ± 0.7	0.757
Glucose (mg/dL)	98 ± 15	94 ± 10	99 ± 6	0.800
Insulin (IU/L)	29 ± 10	30 ± 12	35 ± 12	0.757
CRP (mg/mL)	4.2 (3.7)	4.9 (2.8)	4.7 (2.1)	0.909†
IL-6 (pg/mL)	445 ± 60	474 ± 53	443 ± 51	0.757
TNF (pg/mL)	0.97 ± 0.27	1.24 ± 0.39	1.01 ± 0.35	0.330
VEGF (pg/mL)	137 ± 23	142 ± 30	163 ± 13	0.087
LPS (ng/mL)	721 ± 266	1221 ± 728	1211 ± 490	0.087
vWF (ng/mL)	83 ± 5	84 ± 6	84 ± 7	0.976
TM (ng/mL)	4.1 ± 0.6	4.2 ± 0.7	4.3 ± 0.8	0.917

LPD - Low probiotic dose, HPD - High probiotic dose; †-Kruskal-Wallis test, ANOVA otherwise; Q - FDR adjusted p value; BMI, body mass index; TBW, total body water; HR, heart rate; SBP, systolic blood pressure; DBP, diastolic blood pressure; PWA Alx, pulse wave analysis augmentation index; PWA AP, pulse wave analysis aortic pressure; PWA PP, pulse wave analysis pulse pressure; PWA SP, pulse wave analysis systolic pressure; PWV, pulse wave velocity; LDL-C, low-density lipoprotein cholesterol; HDL-C, high-density lipoprotein cholesterol; TG, triglycerides; CRP, C-reactive protein; Il-6, interleukin-6; TNF, tumor necrosis factor-alpha; VEGF, vascular endothelial growth factor; LPS, lipopolysaccharide; vWF, von Willebrand factor; TM, thrombomodulin. The data are the arithmetic mean ± SD or median (interquartile range).

### Effect of Probiotic Supplementation on the Composition and Function of Gut Microbiome

**Figure 1:**
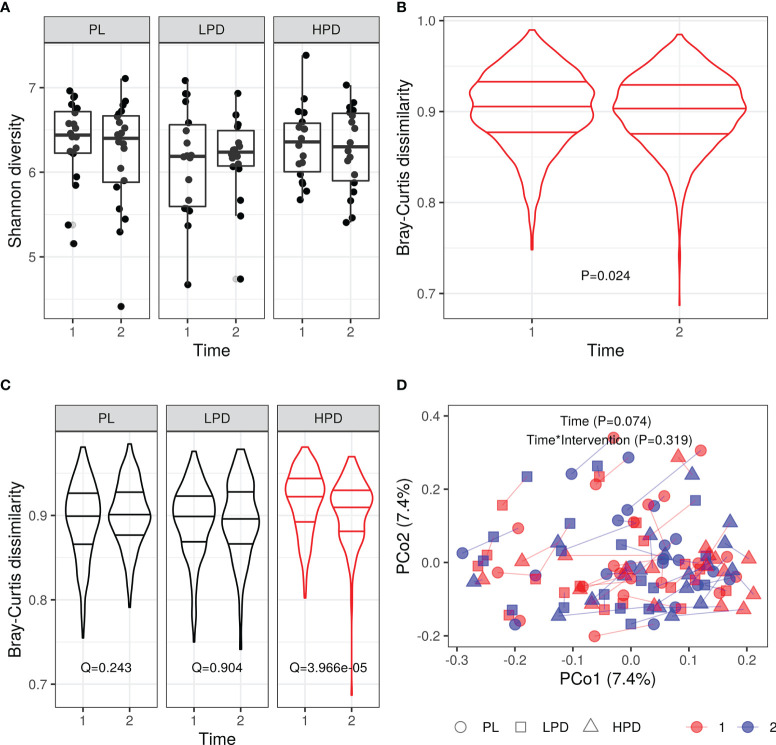
Probiotic intervention and its impact on the gut microbiota diversity **(A)** Shannon diversity (Q = 0.507 PL vs. LPD vs. HPD, Q = 0.639 PL vs. LPD + HPD); **(B)** beta diversity: all intersample Bray-Curtis distances per time point, P = 0.024; **(C)** Beta diversity (intersample Bray-Curtis distances per intervention and time point); **(D)** Principal coordinate analysis (PCoA) based on the Bray-Curtis distances: same donor samples (two time points are connected by line); horizontal lines in violin plots represent quartiles and median.

We did not find any significant changes in alpha diversity throughout the study, and there were no intervention-dependent differences in changes to alpha diversity (**Supplementary Table 1** and **Figure 1A** for the Shannon likelihood ratio test Q = 0.507). In contrast, we observed significant differences in the overall intersample Bray-Curtis distances between time points (Wilcoxon rank-sum test P = 0.024, **Figure 1B**) and intervention-specific distances (**Figure 1C**, Bray-Curtis intersample distance per intervention group and time point - likelihood ratio test for Intervention*Time interaction, P = 0.0004, *post-hoc* Wilcoxon rank-sum test, time point 1 vs. time point 2, PL, Q = 0.243; LPD, Q = 0.904; HPD, Q = 3.97e-05). However, it was not paralleled by corresponding intervention-specific differences in within-sample (same subjects) Bray-Curtis distances (Median (IQR) PL: 0.46 (0.08), LPD: 0.47 (0.14), HPD: 0.49 (0.13), Kruskal-Wallis, P = 0.445). In line with this, permutational analysis of variance (PERMANOVA) using Bray-Curtis distance between samples in the original space (stratified by subject) did not reveal a significant time (P = 0.074) and time by intervention interaction (P = 0.319) effects on sample dissimilarity (**Figure 1D**). Thus, the decrease in Bray-Curtis dissimilarity after 12 weeks (**Figure 1C**) suggests shaping of the overall gut microbiota by a high-dose probiotic intervention. Additionally, between-subjects Bray-Curtis distances at baseline were significantly higher than the same donor (within-subject) dissimilarities between time points (median (IQR) between-subjects: 0.91 (0.05), within-subjects: 0.48 (0.11), Wilcoxon rank-sum test unpaired P < 0.0001). Overall, the individual microbiome stability was not affected by probiotic administration.

Here, we examined the taxa of the gut microbiome and predicted functional modules for changes in abundance over time between the intervention groups. Twenty-seven percent of features (1,253 out of 4,716) could be classified down to the species level (83 distinct species, **Supplementary Table 2**).We did not observe relevant shift in bacterial abundance over time, and abundance shifts did not depend on the type of treatment when probiotic groups were considered separately (PL vs LPD vs HPD, **Supplementary Figure 2**). For the PICRUSt2 predicted MetaCyc pathways, we observed a stronger yet insignificant effect in the HPD group (**Supplementary Figure 3**). In addition to the general linear model framework, we also used the random forest classification with SHAP between the time points in different groups to identify the most important features (bacteria) that changed during the intervention. When only the HPD group was considered, the average 3-fold cross-validation ROC and PR curves AUC scores were 0.55 and 0.62, respectively. Merging the HPD group with LPD improved the average ROC AUC score (0.66) but not the PR AUC (0.66) while merging the LPD with the PL group markedly worsened both scores (**Supplementary Figure 4**).

### Associations of Gut Microbiota With Probiotic Intervention

**Figure 2:**
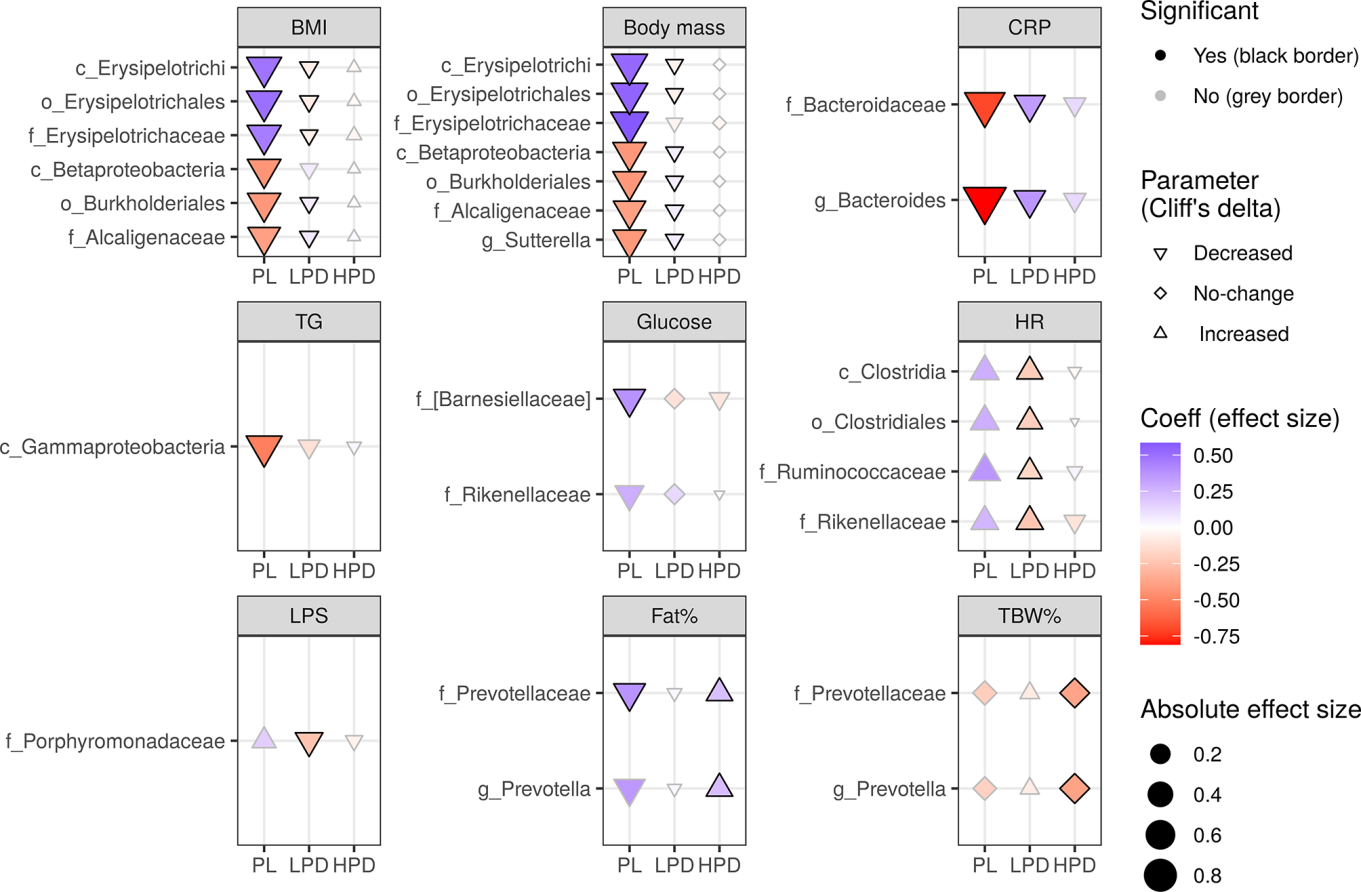
The impact of probiotic intervention on the effect of baseline bacterial abundance on changes in cardio-metabolic parameters during the study. The shapes of the points (diamond, triangle point down, triangle point up) were mapped to the sign of Cliff’s effect size indicating no change, decrease, or increase in value, respectively [the significance of these changes was not the aim here as it was already presented in the previous papers (Szulińska et al., 2018a; Szulińska et al., 2018b)]. The strength and direction of the intervention effect, as indicated by standardised coefficients, are represented by the size (absolute value) and color of the points. A change in any parameter with time, either increase (triangle point up) or decrease (triangle point down), can be counteracted (red triangle point up, blue triangle point down) or enhanced (blue triangle point up, red triangle point down) by baseline bacterial abundance. A change may also be induced by the baseline microbiota (diamonds). Probiotics can have an opposite effect (if they reverse the sign of the coefficient, color changes from red to blue or from blue to red) or an analogous effect (if the sign of the coefficient and color remains the same, the effect is strengthened). The significance of the three-way interaction of time by pre-treatment (or follow-up) abundance by the intervention was first tested by a likelihood ratio test (LRT) of nested models (for the P value indicating whether the overall set of interactions was significant) followed by a Satterthwaite’s degrees of freedom method (for individual P values). Significant individual P values (< 0.05) accompanying fixed effects of the interactions are represented by a black border. LRT P values were used to compute the false discovery rate (Q values) within parameters and separately for each taxonomic level. Only taxa with Q < 0.05 are shown.

To examine whether the probiotic intervention has the potential to modulate the effect of gut microbiota on changes in cardiometabolic parameters (time by microbiota interaction), a set of linear mixed-effects models were implemented in which the pre-treatment and post-treatment abundance (as time-invariant covariates), type of intervention, and time, were included. **Figure 2** summarizes the impact of the probiotic intervention on effects of the pre-treatment microbiota (separated into various taxonomic levels) on changes in parameters during the study as identified by significant three-way interactions (a time by baseline microbiota abundance by intervention) using the likelihood ratio test (LRT) followed by FDR adjustment. In total, there were 26 significant (FDR-adjusted) three-way interactions (**Figure 2**): six for the BMI (e.g. class Erysipelotrichi, LRT Q = 0.0006), six for the body mass (e.g. class Betaproteobacteria, LRT Q = 0.002), four for the HR (e.g. class Clostridia, LRT Q = 0.004), two for the CRP (e.g. family Bacteroidaceae, LRT Q = 0.003), glucose (e.g. family Rikenellaceae, LRT Q = 0.002), Fat% (e.g. family Prevotellaceae, LRT Q = 0.002), TBW% (family Prevotellaceae, LRT Q = 0.0008), and one for the TG (class Gammaproteobacteria, LRT Q = 0.006), and LPS (family Porphyromonadaceae, LRT Q = 0.002).

Specifically, the time by baseline microbiota interaction (i.e. an effect of the baseline microbiota on parameter changes) was modified by probiotics in two ways – the low probiotic dose had overall an opposite effect to that of placebo (BMI, body mass, CRP, HR), whereas the high probiotic dose exhibited mostly no or an analogous (Fat%, TBW%) effect to that observed in the PL group. For example, in the PL, increasing abundance of the family Erysipelotrichaceae **(**and its higher level ranks up to the class Erysipelotrichi), counteracted a decrease in BMI and body mass (as evidenced by the positive standardised coefficients β = 0.49 and β = 0.53, respectively), which was reversed in the LPD (as evidenced by negative standardised coefficients β = -0.08 and β = -0.06, respectively). A similar interaction involved the family Alcaligenaceae (and its higher level ranks up to the class Betaprotecobacteria); however, increasing abundance enhanced a decrease in BMI and body mass (as evidenced by negative β = -0.40 and β = -0.40, respectively), which was reversed in the LPD (as evidenced by positive β = 0.08 and β = 0.07, respectively). CRP, HR, and LPS were affected similarly by a three-way interaction involving Bacteroides and Bacteroidaceae (CRP), Ruminococcaceae, Clostridiales, and Clostridia (HR), and Porphyromonadaceae (LPS). In addition to the opposite effects, probiotics (administered at higher doses) exhibited analogous effects by strengthening the effect of the pre-treatment abundance of Prevotellaceae on TBW% (LRT Q = 0.012) and Fat% (LRT Q = 0.032) observed in the placebo group. Remarkably, only the LPS and glucose changes were shown to depend on probiotic intervention in our previous study (Szulińska et al., 2018b).

**Figure 3:**
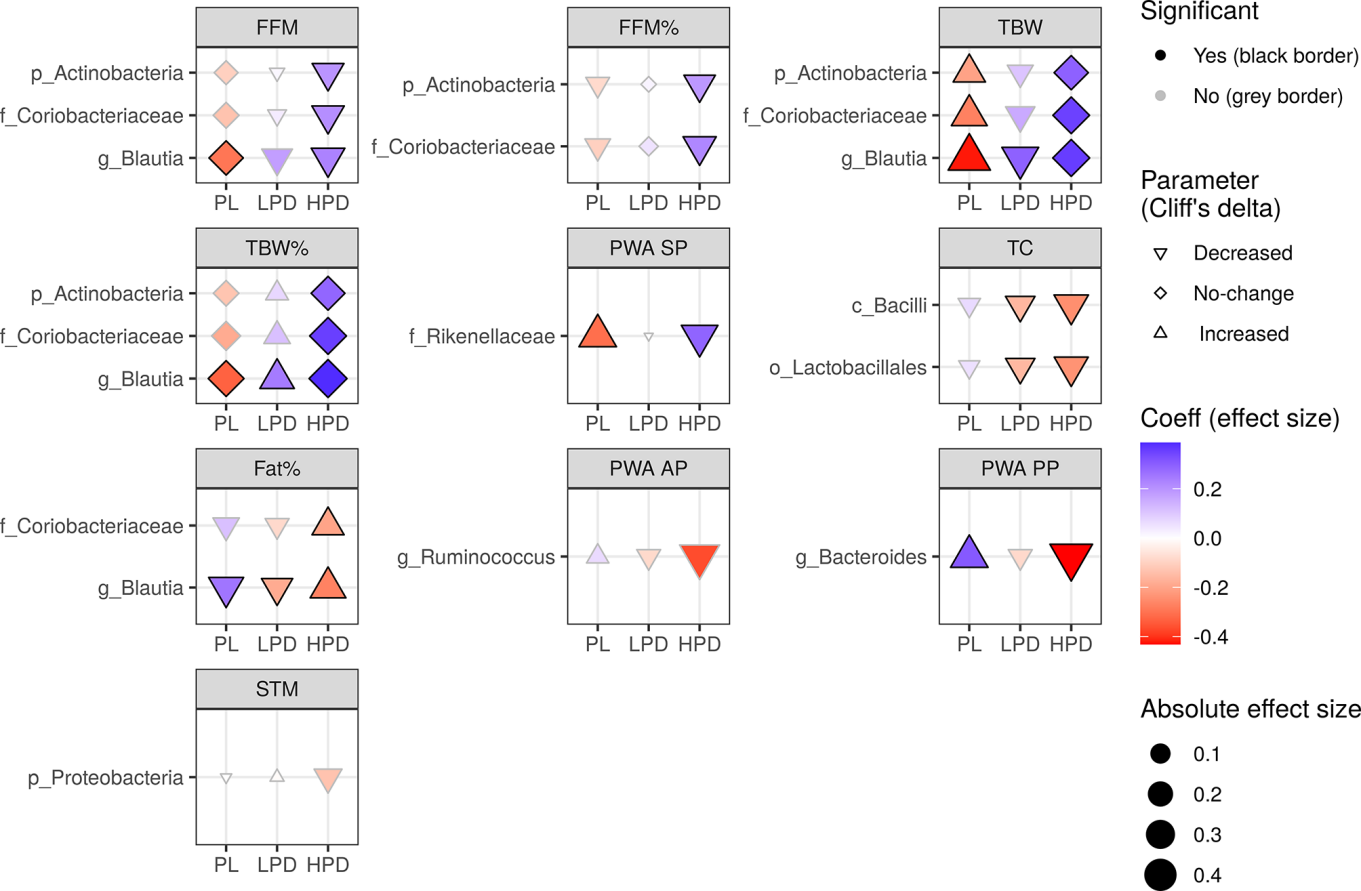
The impact of probiotic intervention on the effect of follow-up bacterial abundance on changes in cardio-metabolic parameters during the study. The shapes of the points (diamond, triangle point down, triangle point up) were mapped to the sign of Cliff’s effect size indicating no change, decrease, or increase in value, respectively. The strength and direction of the intervention effect, as indicated by standardised coefficients, are represented by the size (absolute value) and color of the points. A change in any parameter with time, either increase (triangle point up) or decrease (triangle point down), can be counteracted (red triangle point up, blue triangle point down) or enhanced (blue triangle point up, red triangle point down) by follow-up bacterial abundance. A change may also be induced by the follow-up microbiota (diamonds). Probiotics can have an opposite effect (if they reverse the sign of the coefficient, color changes from red to blue or from blue to red) or an analogous effect (if the sign of the coefficient and color remains the same, the effect is strengthened). The significance of the three-way interaction of time by pre-treatment (or follow-up) abundance by the intervention was first tested by a likelihood ratio test (LRT) of nested models (for the P value indicating whether the overall set of interactions was significant) followed by a Satterthwaite’s degrees of freedom method (for individual P values). Significant individual P values (< 0.05) accompanying fixed effects of the interactions are represented by a black border. LRT P values were used to compute the false discovery rate (Q values) within parameters and separately for each taxonomic level. Only taxa with Q < 0.05 are shown.

Although we found no relevant shifts in the microbiota during the study (**Supplementary Figure 2**), we also examined whether the probiotics could be involved in altering the effect of post-treatment bacterial abundance on parameter changes, that is, whether a three-way interaction by time by post-treatment abundance by intervention exists, after controlling for the pre-treatment abundance. In total, there were 19 significant after FDR adjustment three-way interactions (**Figure 3**): three for the FFM, TBW, and TBW% (e.g. Coriobacteriaceae, LRT Q = 0.016, Q = 0.001, Q = 0.003, respectively), two for the FFM%, Fat% (e.g. Coriobacteriaceae, LRT Q = 0.014, Q = 0.046, respectively), TC (e.g. Lactobacillales, LRT Q = 0.038), and PWA SP (e.g. Rikenellaceae, LRT Q = 0.040), PWA PP (e.g. Bacteroides, LRT Q = 0.021), and PWA AP (e.g. Ruminococcus, LRT Q = 0.049). Here, the impact of probiotics was stronger in the HPD group, which was opposite to that observed in the PL group. For example, the effects of Blautia on FFM, TBW, TBW%, and Fat% were reversed by administration of a higher dose of probiotics. For example, the increasing abundance of Blautia counteracted a decrease in Fat% in the PL (β = 0.26), and this effect was reversed in the HPD group (β = -0.27). Moreover, HPD favorably affected the influence of Actinobacteria and Coriobacteriaceae on FFM (PL group - β = -0.11, HPD group - β = 0.20) and TBW (PL group - β = -0.21, HPD group - β = 0.29). From the measurements outlined here, only PWA SP changes differed between the interventions, as reported previously (Szulińska et al., 2018a).

### Gut Microbiota Did Not Vary in Therapy Responders and Non-Responders

Responders were defined based on Cohen’s effect size and partition around medoids (PAM) clustering, as described in the Methods section. Among the six sets of variables taken into account (see *Materials and Methods*), only blood pressure (Cohen’s standardised differences of the systolic blood pressure and diastolic blood pressure) allowed for reliable grouping of observations (average silhouette width of 0.34). The silhouette plots for all sets of parameters are shown in **Supplementary Figure 5**. Characteristics of the responders and non-responders are shown in **Table 2**. Standardised differences in systolic and diastolic blood pressures in responders and non-responders are shown in **Supplementary Figure 6**. Grouping of blood pressure response was driven primarily by DBP change which was (median (IQR) Cohen’s d) -0.66 (0.93) in responders (n = 21) and 0.53 (0.73) in non-responders (n = 35).

**Table 2:** Baseline characteristics in responders and non-responders with respect to diastolic blood pressure change.

	Responders (n = 21)	Non-responders (n = 35)	Q
Age (years)	59 ± 8	55 ± 7	0.717
Body mass (kg)	95 ± 9	92 ± 13	0.976
BMI (kg/m2)	36 ± 4	36 ± 4	0.943
Subcutaneous fat area (cm2)	300 ± 58	280 ± 64	0.943
Visceral fat area (cm2)	228 ± 57	213 ± 58	0.717
Waist circumference (cm)	111 ± 7	109 ± 9	0.915
Fat mass (kg)	49 ± 9	47 ± 11	0.976
Fat %	53 (8)	53 (8)	0.943†
Fat free mass (kg)	46 ± 6	44 ± 7	0.717
Fat free %	46 ± 7	47 ± 8	0.717
TBW (liters)	34 (4)	34 (5)	0.717†
HR (bpm)	73 ± 8	76 ± 8	0.269
SBP (mmHg)	132 (23)	138 (7)	0.761†
DBP (mmHg)	78 ± 8	84 ± 7	0.717
PWA Alx	36 ± 12	31 ± 11	0.976
PWA AP (mmHg)	15 ± 8	13 ± 7	0.943
PWA PP (mmHg)	42 ± 9	44 ± 9	0.943
PWA SP (mmHg)	125 ± 11	131 ± 11	0.717
PWV (m/s)	7.2 ± 1.0	7.1 ± 1.0	0.717
Total cholesterol (mg/dL)	208 ± 32	218 ± 43	0.717
LDL-C (mg/dL)	126 ± 35	124 ± 43	0.943
HDL-C (mg/dL)	53 ± 10	56 ± 12	0.717
TG (mg/dL)	146 (69)	154 (59)	0.717†
Uric acid (mmol/L)	5.4 (0.8)	5.7 (1.0)	0.717†
Glucose (mg/dL)	98 ± 15	96 ± 8	0.717
Insulin (IU/L)	27 ± 9	34 ± 12	0.717
CRP (mg/mL)	4.7 (2.9)	4.6 (3.6)	0.909†
IL-6 (pg/mL)	453 ± 54	454 ± 57	0.717
TNF (pg/mL)	0.94 (0.45)	1.02 (0.34)	0.505†
VEGF (pg/mL)	147 (15)	154 (25)	0.018†
LPS (ng/mL)	876 (756)	908 (667)	0.145†
vWF (ng/mL)	83 ± 6	84 ± 6	0.976
TM (ng/mL)	4.2 ± 0.5	4.2 ± 0.8	0.923

LPD - Low probiotic dose, HPD - High probiotic dose; †-Kruskal-Wallis test, ANOVA otherwise; Q - FDR adjusted p value; BMI, body mass index; TBW, total body water; HR, heart rate; SBP, systolic blood pressure; DBP, diastolic blood pressure; PWA Alx, pulse wave analysis augmentation index; PWA AP, pulse wave analysis aortic pressure; PWA PP, pulse wave analysis pulse pressure; PWA SP, pulse wave analysis systolic pressure; PWV, pulse wave velocity; LDL-C, low-density lipoprotein cholesterol; HDL-C, high-density lipoprotein cholesterol; TG, triglycerides; CRP, C-reactive protein; Il-6, interleukin-6; TNF, tumor necrosis factor-alpha; VEGF, vascular endothelial growth factor; LPS, lipopolysaccharide; vWF, von Willebrand factor; TM, thrombomodulin. The data are the arithmetic mean ± SD or median (interquartile range).

Blood pressure responders (BPR) were found in all study arms; however, there was no significant over-representation of the BPR in the arms of the study. The distribution of BPR in the arms of the study was as follows: 50.0%, 27.8%, and 33.3% in the PL, LPD, and HPD groups, respectively (Fisher’s exact test P = 0.354). To explore whether the baseline microbiota could have an impact on responses, first, the overall bacterial composition at different taxonomic levels was compared between responders and non-responders using PERMANOVA based on the Bray-Curtis dissimilarities (**Supplementary Figure 7**); second, the bacterial abundance was compared between responders and non-responders while controlling for the type of intervention using a general linear model framework. We found no response-specific microbiota signature, which suggests that the response was not modulated by the gut microbiota (**Supplementary Figure 8**).

## Discussion

This study presents a multidirectional and comprehensive analysis of the effects of administering multispecies probiotics on the microbiota of postmenopausal women with obesity. Previously published studies have shown the beneficial effects of this probiotic combination on various metabolic markers (Sabico et al., 2017; Szulińska et al., 2018b; Sabico et al., 2019; Majewska et al., 2020) and vascular function (Szulińska et al., 2018a), and it is also known that microbiota alterations are observed in metabolic disorders (Cani et al., 2021). Therefore, verifying this research hypothesis would explain its beneficial metabolic effects. It should be noted that the mechanisms underlying the physiological or clinical effects of probiotic administration are still unknown and of great interest to researchers (Wieërs et al., 2019).

We observed that the metabolic and physiological effects of PB were associated with the microbiota composition analyzed at baseline (pre-treatment) and endpoint (post-treatment). Interestingly, when these effects were compared to the control group, the nature of the associations was often opposite. This observation suggests that a probiotic intervention may modify the effect of microbiota on individual biochemical and physiological parameters, regardless of changes in microbiota composition and metabolic function over time. Similar observations were made by McNulty et al., who found a change in the metabolic functions of the microbiota under the influence of probiotics, despite no change in its composition (McNulty et al., 2011). Our data suggest that probiotics may work without altering the composition of the microbiota but by changing its function, which is considered more stable (Human Microbiome Project Consortium, 2012). We did not also observe unequivocal relationships between the effect of probiotics and the dose. It should also be stressed that changes in bacterial function did not always follow a direction favourable to the expected effect of probiotic administration. However, based on the metadata available, it can be concluded that the eventual effect of probiotics on metabolism was beneficial for the patients. The most noteworthy relationship was observed between baseline microbiota composition and body weight changes. The increased abundance of bacteria of the class Erysipelotrichi and belonging to its lower taxonomic groups counteracted the decrease in body mass and BMI observed in patients in the control group. Interestingly, in the LPD group, this effect was opposite and the bacteria appeared to act synergistically with probiotics on body mass and BMI. In the HPD group, no association between bacteria and these anthropometric parameters was observed. Thus, it can be said that probiotics have a positive influence on the effects of the Erysipelotrichi class of bacteria on body weight. In contrast, the increased abundance of Betaproteobacteria class strengthened BMI and body weight decrease in the control group, and this association was alleviated in the group of patients treated with LDP and not observed in the HDP group. This may indicate that the beneficial effect of this bacterial group on body weight was reduced under the influence of probiotic therapy. However, from a practical point of view, the most important is the final effect of the probiotic on a given physiological parameter. The association of the family Erysipelotrichaceae with metabolic disorders has been known for a long time and has been confirmed in many publications (Kaakoush, 2015). Turnbaugh et al. observed an increased abundance of species belonging to Erysipelotrichaceae in diet-induced obesity (Turnbaugh et al., 2008). Zhang et al. observed an association between Erysipelotrichaceae and obesity (Zhang et al., 2009). Additionally, supplementation of anti-obesity agents inhibits the growth of Erysipelotrichaceae (Etxeberria et al., 2015). Reports concerning bacteria belonging to the Betaproteobacteria class are inconsistent. Betaproteobacteria levels are reduced in children with obesity (Quiroga et al., 2020). However, another study (Volynets et al., 2017) revealed that the high fat fraction of the Western-style diet causes an increase in Burkholderiales, and this Betaproteobacteria order correlated with weight gain. This also confirms the correlation between specific Burkholderiales and weight gain in psychiatric patients (Bahr et al., 2015). In contrast, the relative abundance of the family Alcaligenaceae significantly decreased in obesity (Palmas et al., 2021). In our study, the genus *Suturella* was shown to enhance a decrease in body weight in women taking a placebo, and this effect was undermined in the LDP group, which is an undesirable effect for probiotic therapy. Previously, it was described that this type of bacteria was enriched in obese individuals (Chen et al., 2021). It is important to note that our observations may explain the often conflicting data on the effect of the abundance of different bacteria on body weight. Perhaps not only probiotics but dietary habits, exercise, or xenobiotics may alter bacterial functions, which should be taken into account in microbiome studies and the subject of multivariate analyses. Interestingly, the family Prevotellaceae and genus *Prevotella* were positively associated with Fat% in both the placebo and HDP groups, despite the opposite directions of changes associated with the type of intervention. *Prevotella* is a strong marker of obesity (Duan et al., 2021) and its function was not affected by probiotic therapy in our study. Barnesiellaceae abundance in the control group was significantly positively associated with end-point glucose concentration, and this unfavorable effect was not observed in groups receiving probiotics. These bacteria are known to play a role in glycemic control (Hughes et al., 2021).

A synergistic association between Bacteroidaceae and CRP was observed in women in the control group, which was alleviated in the LDP group. Bacteroidaceae families significantly increased during the initiation (Liu et al., 2016) and decreased in the preclinical phase of inflammatory diseases (Rogier et al., 2017). The abundance of Porphyromonadeceae, which is associated with periodontal disease (Kharitonova et al., 2021), was synergistically associated with LPS concentration in the LPD group, which is favorable for patients.

Changes in the association between bacteria and physiological parameters according to the type of intervention were more unequivocal at the end of the study. In the groups receiving probiotics, a beneficial association was observed between phylum Actinobacteria, family Coriobacteriaceae, and genus *Blautia* and different parameters of body composition. Thus, it can be said that probiotic therapy supports the beneficial effects of these bacteria on body weight. Actinobacteria are involved in metabolic health, and the abundance of this family is associated with the consumption of dietary fibre and the production of SCFAs (Hustoft et al., 2017; Binda et al., 2018). Therefore, it may have anti-inflammatory properties and improve the intestinal barrier integrity (Vinolo et al., 2011). Actinobacteria were more abundant in Western (Illescas et al., 2021) and high sugar (Moreira Júnior et al., 2021) diets and were found in higher concentrations in obese individuals (Turnbaugh et al., 2009). An increased abundance of Actinobacteria was also associated with type 2 diabetes in urban Africa (Doumatey et al., 2020). In contrast, another study showed that Actinobacteria levels decreased in obese individuals (Duan et al., 2021). The family Coriobacteriaceae plays important metabolic roles such as the conversion of bile acid, steroids, and phytoestrogens, glucose homeostasis, and lipid metabolism, and is associated with good metabolic health in overweight and obese populations (Kim et al., 2020). This family has been investigated in metabolic diseases (Clavel et al., 2014; Liu et al., 2018), although our knowledge of the underlying molecular mechanisms is limited. Blautia is involved in the production of butyric and acetic acids (Liu et al., 2015) which decreases obesity by regulating G-protein coupled receptors (GPR) 41 and 43 (Kimura et al., 2011; Kimura et al., 2013). Its abundance is inversely associated with visceral fat accumulation (Ozato et al., 2019) and diabetes (Larsen et al., 2010; Murri et al., 2013), and members of the Bacteroidales order, Rikenellaceae, and Bacteroides, which can harbor butyrate-producing members, were also negatively correlated with blood pressure (Gomez-Arango et al., 2016). A negative correlation between Bacteroides and blood pressure, body weight, and fat mass have been reported (Munukka et al., 2012; Queipo-Ortuño et al., 2012). Therefore, supplementation with bacteria belonging to this genus may improve multiple clinical parameters. However, based on the results obtained and the results reported in the responders, it cannot be fully concluded that baseline microbiota can be considered as a predictor of response to the intervention, and microbiota at the endpoint might be used as an intervention marker.

We did not confirm the effect of probiotic administration on alpha and beta diversity. Although we observed a decrease in beta diversity measured by Bray-Curtis dissimilarity for the whole study group, which is driven by higher doses of probiotics, the intrasample analysis indicated that this change was not driven by alterations of microbiota in the same patients.

Similarly, a fine-grade taxonomic and functional module analysis did not show significant changes in these features over time, and the probiotics did not contribute to these changes. It should be noted that the PICRUSt2 analysis indicates a greater effect of HDP intake on metabolic pathways, but probably due to the small sample size of the group, the observed values were not statistically significant.

To identify the key features capable of distinguishing two time points of the study, we also applied random forest models. However, the accuracy of distinguishing the time points 12 weeks apart in the HPD group was low. Merging both probiotic groups (LPD and HPD) resulted in a slight improvement which could be explained by either an increase in the number of data points available or, more importantly, by a similar composition shift in both treatment groups. Although only one genus was common in the two analyses (*Oscillosipraceae UCG-002*), this result indicates the overall homogeneity of changes in the probiotic groups.

The results of previous studies that have analyzed the effects of PB on microbiota are inconclusive. Chahwan et al. (2019) did not observe microbiota alterations after PB administration in patients with depression, while Horvath et al. observed such changes in patients with cirrhosis (Horvath et al., 2020a), but not in patients with T2 DM (Horvath et al., 2020b). In the latter study, a better clinical effect of the probiotic was observed in patients who had an increase in the abundance of *L. brevis*. Similarly, Zhao et al. (2019) observed an association between microbiome alteration after prebiotic administration and improvement in glycemic control Bloemendaal et al. (2021) observed changes in the microbiota of healthy female patients receiving PB, which were insignificant after multiple comparison corrections. It should be emphasized that not all studies utilized compositionality-aware computational methods. Studies in which microbiome datasets are converted to relative abundances may have an unacceptably high rate of false-positive identification of differentially abundant bacteria.

There is currently a contention on whether probiotic treatments can successfully alter microbiota composition (Kristiansen, 1990; Ki Cha et al., 2012). The 16S rRNA sequencing method is not sensitive enough to verify the microbiota alterations induced by probiotic administration (Knight et al., 2018; Puebla-Barragan and Reid, 2019). Furthermore, it is not a prerequisite for a probiotic to confer benefits by significantly changing the gut microbiota of the host (Puebla-Barragan and Reid, 2019). Rather, the health benefit can be accrued through metabolites produced by the probiotic strains as they pass through the intestine (McNulty et al., 2011), and through interactions with the host’s metabolism (Bazanella et al., 2017) and immune system even in healthy adults (Harbige et al., 2016). However, probiotics have been shown to modulate gut microbiota gene expression in the absence of compositional changes, with potential anti-inflammatory effects (Eloe-Fadrosh et al., 2015). This is one potential mechanism by which probiotics may affect metabolic function. Alternatively, probiotic bacterial species may exert an effect on the host directly, as PB has been shown to improve gut barrier function *in vitro* (Hemert and Ormel, 2014). The dosage size of the probiotic may not have been sufficient to be detected in the stool, but it still resulted in the metabolic effects observed in the study. For example, it was shown that probiotic supplementation of *L. rhamnosus GG* at 10^8^ CFU was detectable in only 1 of 10 faecal samples; however, the same strain at a higher dose of 10^12^ CFU was detected in all 10 faecal samples (Saxelin et al., 1995). In this study, we used two daily doses of probiotics, 2.5x10^9^ CFU and 10^10^ CFU, which is above the minimum dose requirement for probiotics without strain-specific claims (Hill et al., 2014) and comparable with other studies analyzing this probiotic combination in metabolic diseases (Sabico et al., 2017; Sabico et al., 2019) and also in studies where the composition of microbiota was analyzed (Chahwan et al., 2019; Horvath et al., 2020a; Horvath et al., 2020b; Bloemendaal et al., 2021). Further research using a range of concentrations in a dose-response study may be warranted to determine the optimal dose. Potentially, a greater dose or longer consumption of probiotics would have produced a detectable change in gut microbiota, as well as further differences in metabolic data between probiotic and placebo groups. In other studies that have evaluated the effect of probiotics on cardiometabolic risk factors in healthy individuals, microbiota analysis is often not performed. In a recently published systematic review and meta-analysis (Skonieczna-Żydecka et al., 2020) concerning the effect of probiotics and synbiotics on risk factors associated with cardiometabolic diseases in healthy people, faecal microbiota composition was tested only in 13 of 61 studies. The NGS technique was used in only four studies. In six studies, the clinical outcomes were associated with microbial changes. In four studies, changes in microbiota were observed despite the lack of clinical efficacy of probiotic treatment. Notably, the results could not be subjected to meta-analysis because of the diverse analytical methods used to analyses the microbiota. Therefore, it was not possible to determine whether the effect of probiotics on cardiovascular risk prevention is related to their effect on the microbiota.

We also assessed whether the composition of the initial microbiota differed between responders and non-responders. This type of analysis provides an opportunity to identify predictors of response to probiotic therapy which is essential for personalized probiotic selection. To date, such a relationship has mainly been observed in response to dietary interventions (Maifeld et al., 2021), although it can also be observed for probiotic use (Zmora et al., 2018). A major problem encountered in such studies is the definition of treatment response. For clinical primary outcomes, this is simpler - the desired clinical effect can be arbitrarily determined (Maifeld et al., 2021). However, the metabolic effects we observed should be treated as a secondary outcome, and a different methodology for qualifying treatment response should be used. We performed a detailed analysis of the response to the intervention based on Cohen’s effect size and machine learning supported clustering techniques, and among the different parameters, we were able to distinguish between responder and non-responder groups only in the case of blood pressure; however, no relationship to baseline microbiota was observed. Further studies on personalized probiotic therapy should include non-invasive methods to determine bacterial colonization as well as metabolomic studies, which will provide more insight into their function.

The study included participants of similar age, sex, body mass, and hormonal status, which allowed the comparison of microbiota composition between subjects (Hasan and Yang, 2019). Moreover, lower, and higher doses of probiotics and the study design (randomized, placebo-controlled, double-blind) provided a great setting for comparative studies of probiotics. The main limitation of this study is the relatively small number of subjects, which affects statistical power, especially when the effects of an intervention on clinical features were investigated. As a result, it is possible to miss some associations between probiotic supplementation and clinical outcomes. However, this does not lessen our confidence in the associations where significant observations were made. The main reasons for the reduced sample size were the strict inclusion and exclusion criteria and limited resources. It should be noted that in the current study, microbiota and clinical metadata were analyzed at only two time points, and we do not know when the change in microbiota function or the biochemical or physiological parameter occurred, for example, microbiota function may have changed on the third and biochemical parameters on the penultimate day of the intervention; therefore, a causal relationship between these phenomena cannot be established. More analyses at additional time points are required to prove this relationship. Furthermore, mechanistic studies such as those using a germ-free mouse model are required to confirm the obtained results. It would be beneficial to compare our results with a similar cohort (Maifeld et al., 2021); however, such head-to-head studies have not been conducted. Other limitations include 16S rRNA sequencing of V1 - V2 regions (in the Polish population the most suitable region is not defined), which provides limited insight into microbiota function (Allaband et al., 2019), and further metabolome and immunome analysis are required. It would also be beneficial to perform mechanistic studies in germ-free mice models to observe the beneficial effect of metabolic activity of probiotics and compare the results with a matched healthy control group.

## Conclusions

1. Probiotic administration may modify the bio-physiological role of the baseline and end-point microbiota, although it does not affect its taxonomic composition.
2. Baseline microbiota composition is not a predictor of therapeutic response to a multispecies probiotic.
3. Further multi-omic and mechanistic studies performed on the bigger cohort of patients are needed to elucidate the cardiometabolic effect of investigated probiotics in postmenopausal obesity.

## Data Availability Statement

The 16S data is available at https://qiita.ucsd.edu/study/description/12903 (public access) and in the EBI ENA database (ebi.ac.uk/ena) with accession number ERP133067.

## Ethics Statement

The studies involving human participants were reviewed and approved by Bioethical Committee of Poznan University of Medical Sciences (No. 871/2015). The patients/participants provided their written informed consent to participate in this study.

## Author Contributions

MK, MS, and PB contributed to the conception and design of the work. MK, IŁ, and PB drafted the paper and substantively revised it. MS, MK-N, and KS-Ż performed the research. MK, TK, and VB performed the bioinformatic analyses. MK, IŁ, TK, and VB analyzed data. All authors read and approved the final manuscript.

## Funding

TK is funded by the Polish National Agency for Academic Exchange grant PPN/PPO/2018/1/00014.

## Conflict of Interest

MK and KS-Ż receive remuneration from probiotic company. IŁ is the probiotic company CEO.

The remaining authors declare that the research was conducted in the absence of any commercial or financial relationships that could be construed as a potential conflict of interest.

## Publisher’s Note

All claims expressed in this article are solely those of the authors and do not necessarily represent those of their affiliated organizations, or those of the publisher, the editors and the reviewers. Any product that may be evaluated in this article, or claim that may be made by its manufacturer, is not guaranteed or endorsed by the publisher.
