# Supplementary material for: Treatment With Multi-Species Probiotics Changes the Functions, Not the Composition of Gut Microbiota in Postmenopausal Women With Obesity: A Randomized, Double-Blind, Placebo-Controlled Study
Source: Front Cell Infect Microbiol. 2022 Mar 11;12:815798. doi: 10.3389/fcimb.2022.815798 (PMC8963764; doi:10.3389/fcimb.2022.815798)
Supplement: Supplementary file 1 [file Image_1.pdf]

1. Supplementary Figures and Tables

1.1 Supplementary Figures

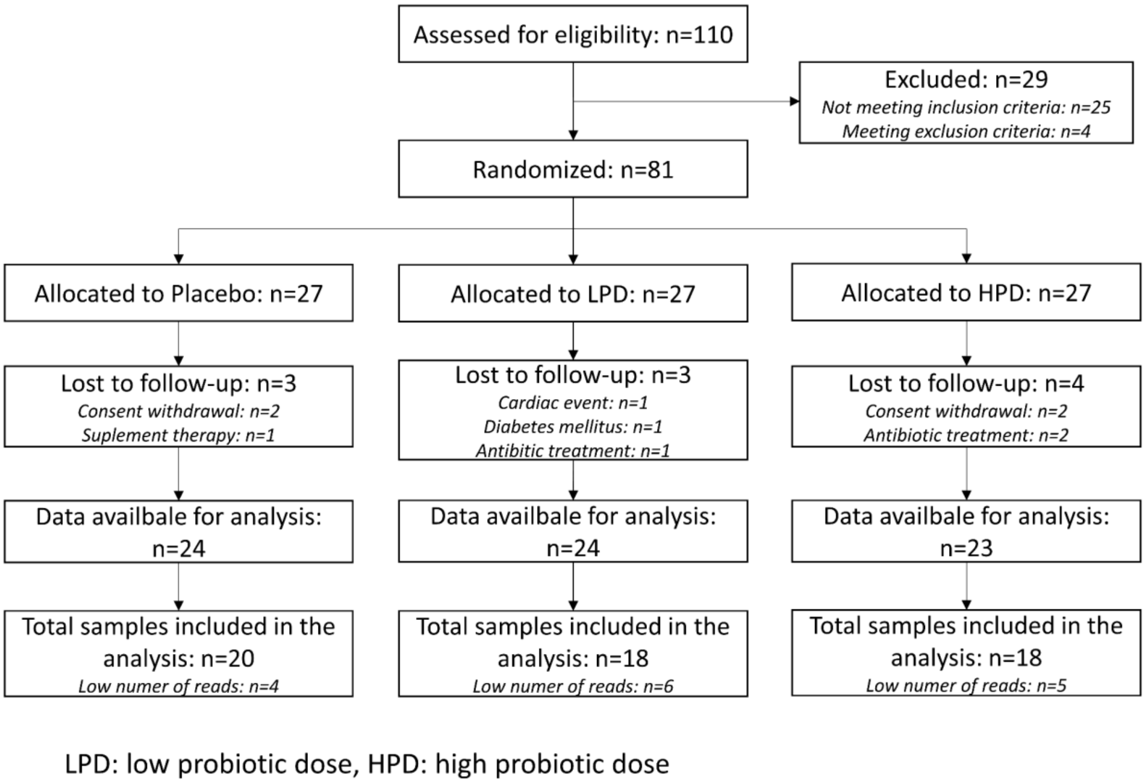

Supplementary Figure 1. Flowchart of the study

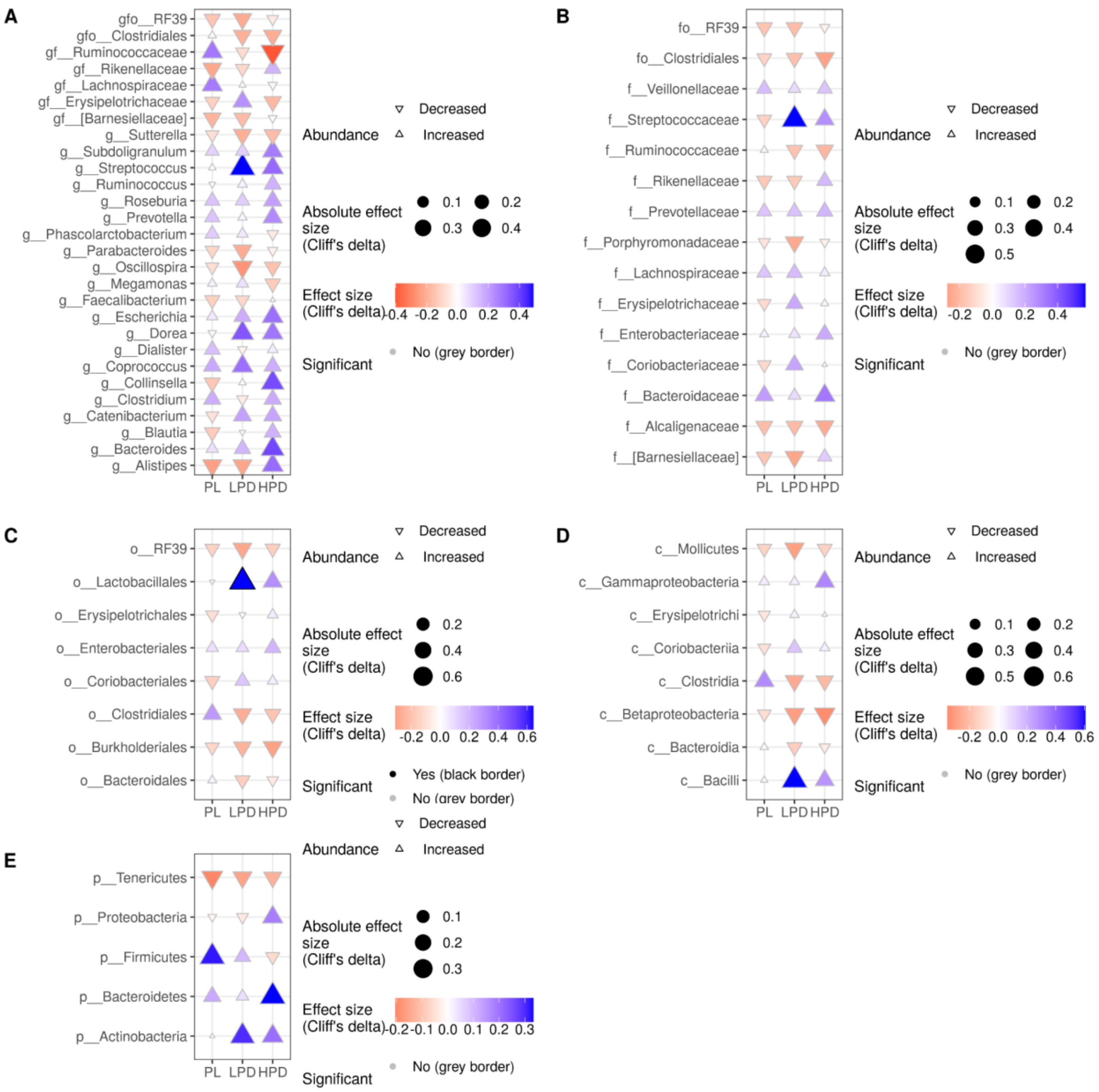

Supplementary Figure 2. Changes of microbiota with time (different taxonomic levels). **(A)** Genus, **(B)** Family, **(C)** Order, **(D)** Class, **(E)** Phylum. The shapes of the points (triangle point down, triangle point up) were mapped to the sign of Cliff's effect size reflecting a difference in abundance between time points. Triangle pointing down and triangle pointing up indicate a decrease and increase in abundance, respectively. The magnitude of Cliff's effect size is represented by the size (absolute value) and color of the points. Significant FDR adjusted P values (< 0.05) of the difference in abundance between time-points are represented by a black border.

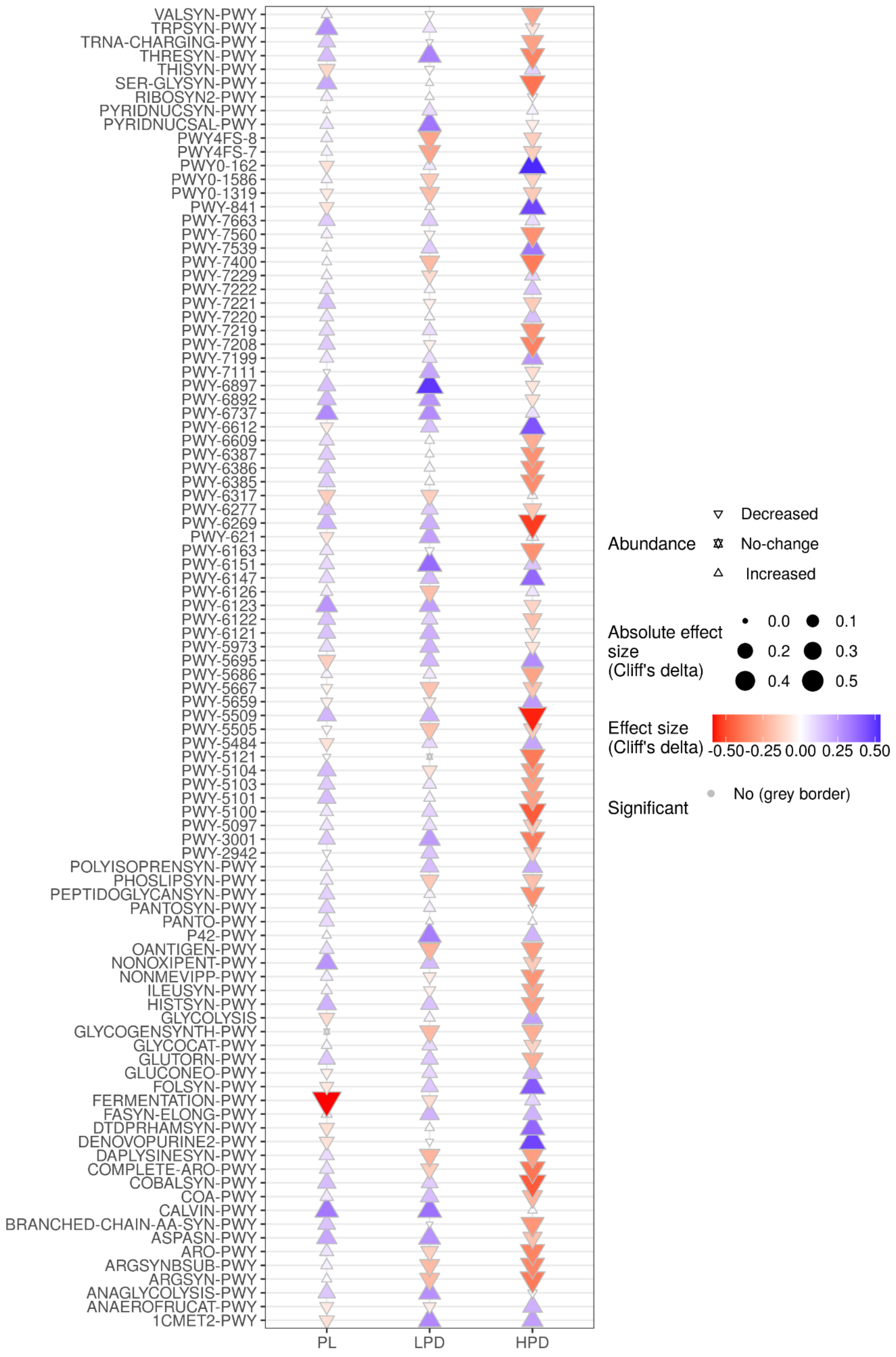
VALSYN-PWY
TRPSYN-PWY
TRNA-CHARGING-PWY
THRESYN-PWY
THISYN-PWY
SER-GLYSYN-PWY
RIBOSYN2-PWY
PYRIDNUCSYN-PWY
PYRIDNUCSAL-PWY
PWY4FS-8
PWY4FS-7
PWY0-162
PWY0-1586
PWY0-1319
PWY-841
PWY-7663
PWY-7560
PWY-7539
PWY-7400
PWY-7229
PWY-7222
PWY-7221
PWY-7220
PWY-7219
PWY-7208
PWY-7199
PWY-7111
PWY-6897
PWY-6892
PWY-6737
PWY-6612
PWY-6609
PWY-6387
PWY-6386
PWY-6385
PWY-6317
PWY-6277
PWY-6269
PWY-621
PWY-6163
PWY-6151
PWY-6147
PWY-6126
PWY-6123
PWY-6122
PWY-6121
PWY-5973
PWY-5695
PWY-5686
PWY-5667
PWY-5659
PWY-5509
PWY-5505
PWY-5484
PWY-5121
PWY-5104
PWY-5103
PWY-5101
PWY-5100
PWY-5097
PWY-3001
PWY-2942
POLYISOPRENSYN-PWY
PHOSLIPSYN-PWY
PEPTIDOGLYCANSYN-PWY
PANTOSYN-PWY
PANTO-PWY
P42-PWY
OANTIGEN-PWY
NONOXIPENT-PWY
NONMEVIPP-PWY
ILEUSYN-PWY
HISTSYN-PWY
GLYCOLYSIS
GLYCOGENSYNTH-PWY
GLYCOCAT-PWY
GLUTORN-PWY
GLUCONEO-PWY
FOLSYN-PWY
FERMENTATION-PWY
FASYN-ELONG-PWY
DTDPRHAMSYN-PWY
DENOVOPURINE2-PWY
DAPLYSINESYN-PWY
COMPLETE-ARO-PWY
COBALSYN-PWY
COA-PWY
CALVIN-PWY
BRANCHED-CHAIN-AA-SYN-PWY
ASPASN-PWY
ARO-PWY
ARGSYNBSUB-PWY
ARGSYN-PWY
ANAGLYCOLYSIS-PWY
ANAEROFRUCAT-PWY
1CMET2-PWY
PL
LPD
HPD
Abundance
Decreased
No-change
Increased
Absolute effect size (Cliff's delta)
0.0
0.1
0.2
0.3
0.4
0.5
Effect size (Cliff's delta)
-0.50
-0.25
0.00
0.25
0.50
Significant
No (grey border)

Supplementary Figure 3. Changes of metabolic pathways with time. The shapes of the points (triangle point down, triangle point up) were mapped to the sign of Cliff's effect size reflecting a difference in abundance between time points. Triangle pointing down and triangle pointing up indicate a decrease and increase in abundance, respectively. The magnitude of Cliff's effect size is represented by the size (absolute value) and color of the points. Significant FDR adjusted P values (< 0.05) of the difference in abundance between time-points are represented by a black border.

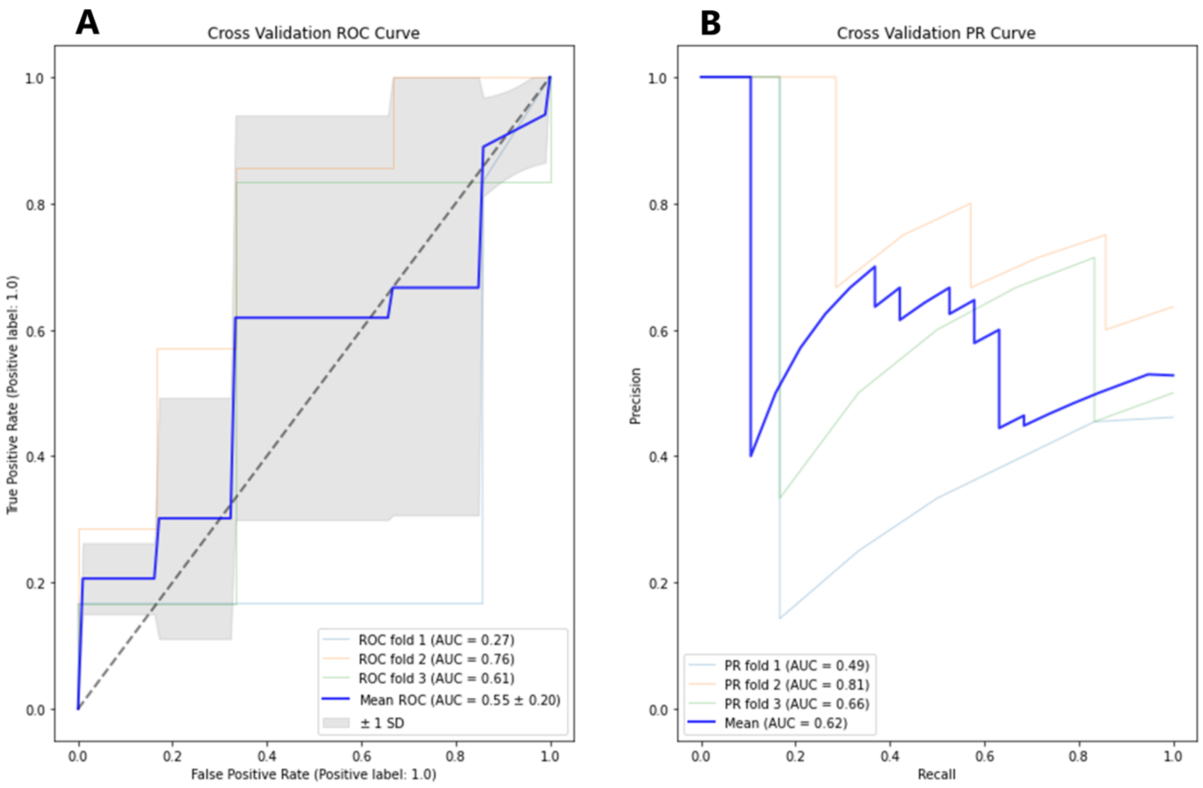

Supplementary Figure 4. Average 3-fold cross-validation ROC **(A)** and PR **(B)** curves. AUC scores for distinguishing time points of the study in the HPD group

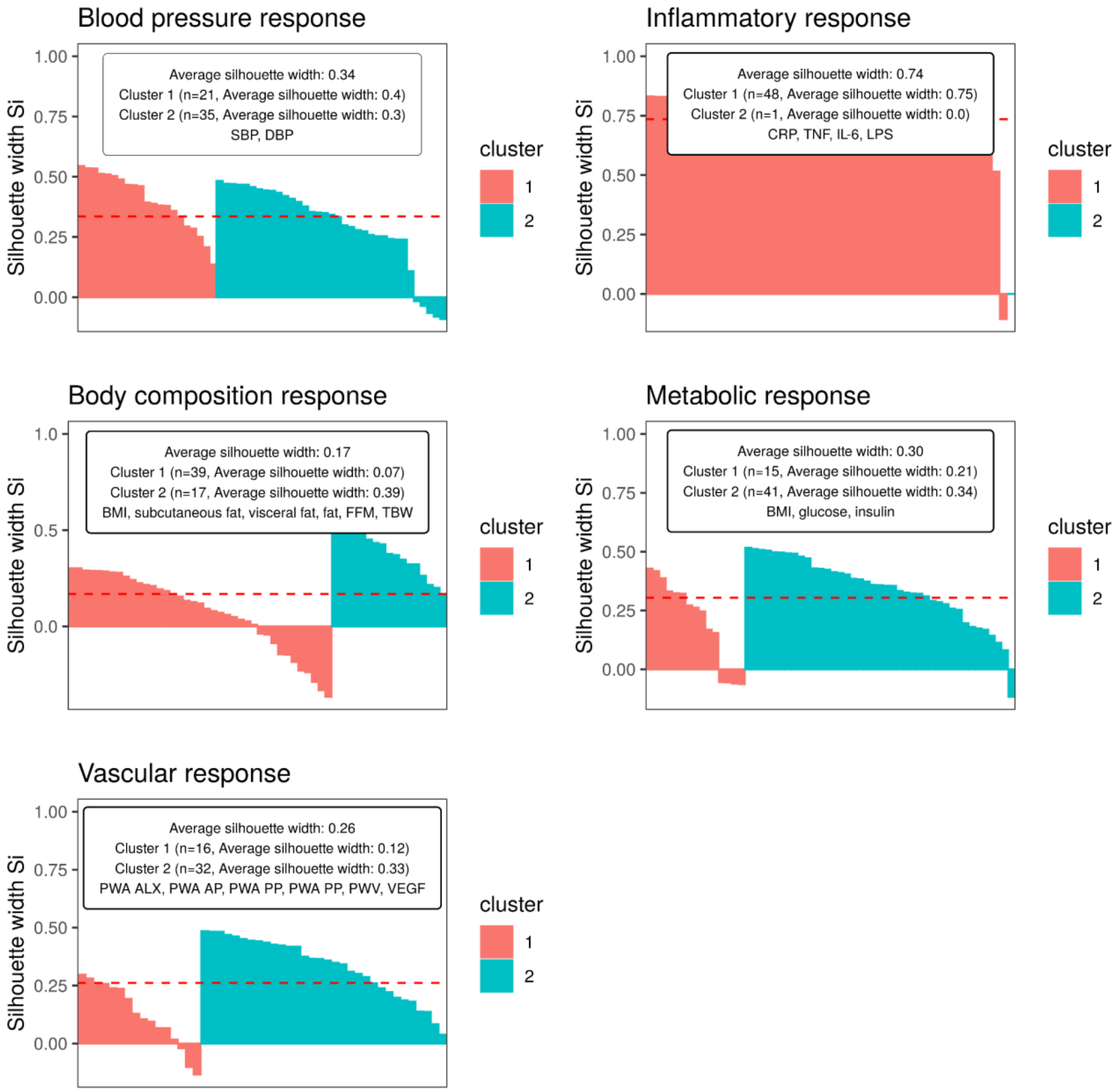

Supplementary Figure 5. PAM (Partition around medoids) silhouette graphs - classification of six sets of variables - each variable represents a standardised difference (Cohen's d between two time points).

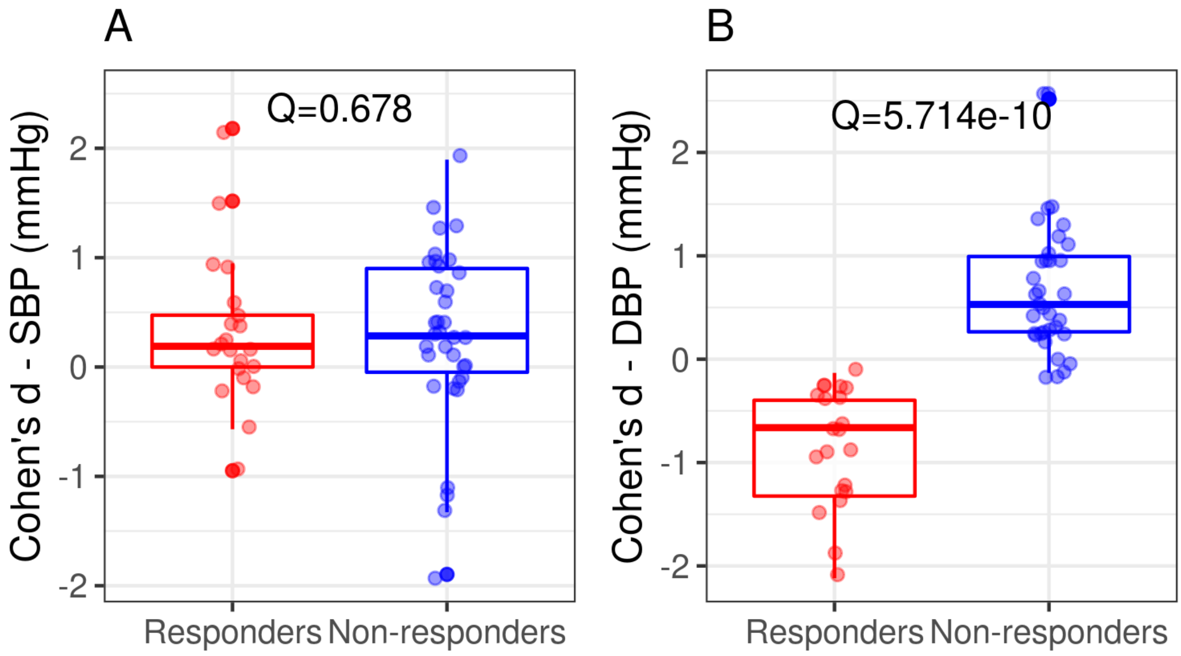

Supplementary Figure 6. Blood pressure - standardised differences between timepoints in responders and non-responders, **(A)** systolic blood pressure, **(B)** diastolic blood pressure. Negative value reflect a decrease over time, Q - FDR adjusted P value

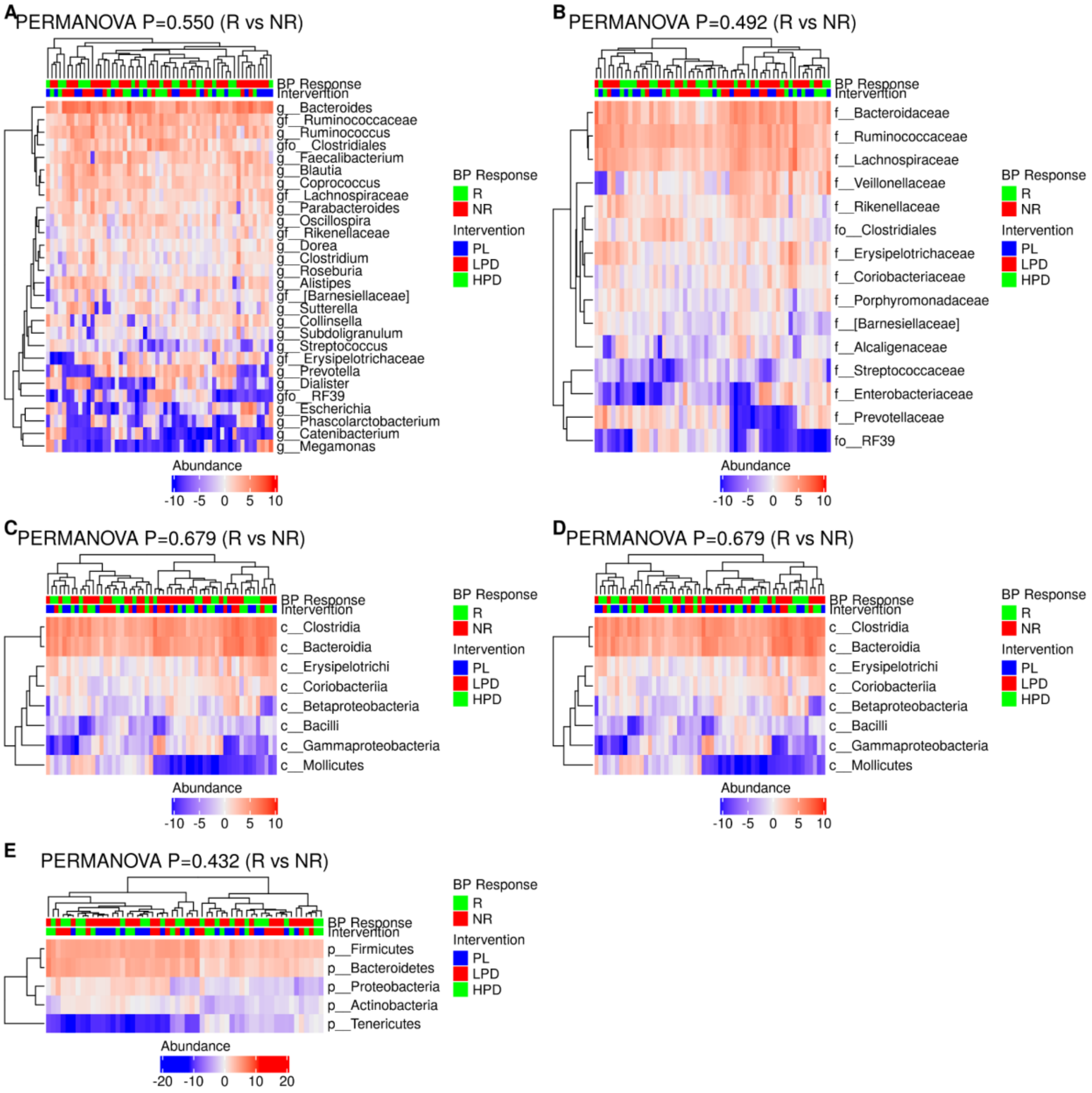

Supplementary Figure 7. Heatmaps showing bacteria in diastolic blood pressure responders and non-responders at various taxonomic levels (PERMANOVA: R vs NR). **(A)** Genus, **(B)** Family, **(C)** Order, **(D)** Class, **(E)** Phylum, PL - placebo, LPD - Low probiotic dose, HPD - High probiotic dose. Clustering (columns and rows) based on the Pearson correlation

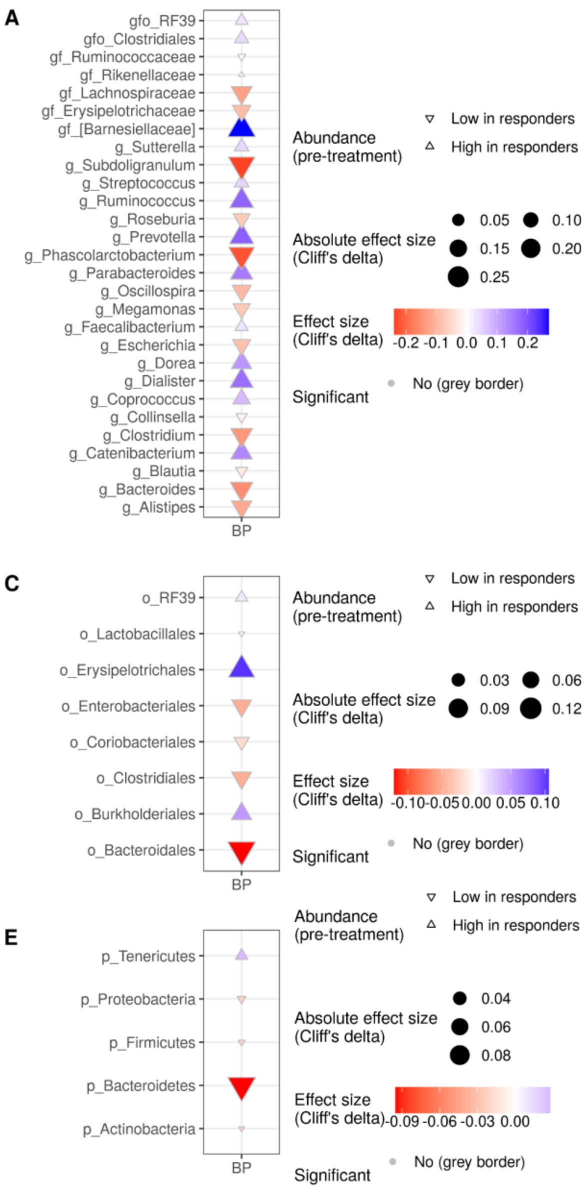

Supplementary Figure 8. Baseline microbiota in diastolic blood pressure (BP) responders and non-responders (different taxonomic levels). **(A)** Genus, **(B)** Family, **(C)** Order, **(D)** Class, **(E)** Phylum. The shapes of the points (triangle point down, triangle point up) were mapped to the sign of Cliff's effect size reflecting a difference in abundance between responders and non-responders. Triangle pointing down and triangle pointing up indicate lower abundance in responders and higher abundance in responders, respectively. The magnitude of Cliff's effect size is represented by the size (absolute value) and color of the points. Significant FDR adjusted P values (< 0.05) of the difference in abundance between responders and non-responders are represented by a black border.

1.2 Supplementary Tables

Supplementary Table 1. Alpha diversity measures by time point and intervention, mean (SD)

Alpha diversity	PL (T1)	PL (T2)	LPD (T1)	LPD (T2)	HPD (T1)	HPD (T2)	P[1]	P[2]	Q[2]
Observed number of sOTUs	259 (48)	253 (64)	238 (52)	228 (44)	259 (51)	265 (58)	0.519	0.507	0.507
Pielou's evenness	0.796 (0.042)	0.782 (0.050)	0.777 (0.057)	0.791 (0.046)	0.792 (0.033)	0.782 (0.044)	0.588	0.264	0.507
Shannon's diversity	6.36 (0.48)	6.22 (0.65)	6.12 (0.64)	6.18 (0.50)	6.34 (0.44)	6.27 (0.50)	0.424	0.493	0.507
Faith's PD	23.6 (3.6)	22.8 (5.6)	21.5 (4.7)	20.9 (4.5)	23.9 (5.1)	24.2 (5.2)	0.360	0.484	0.507

[1] - the effect of time regardless of intervention; [2] - the time point by intervention (PL, LPD, HPD) interaction indicates whether the alpha diversity index change between time points is modified by the type of intervention, Q - FDR adjusted P value; sOTU - suboperational taxonomic unit, PD - Phylogenetic diversity, PL - placebo group, LPD - low probiotic dose, HPD - high probiotic dose, T1, T2 - time points

Supplementary Table 2. Species relative abundance at baseline

Species	Abundance	Total	Frequency (%)
Faecalibacterium prausnitzii	19854	155265	12.787
Bacteroides uniformis	17718	155265	11.411
Bacteroides plebeius	14050	155265	9.049
Prevotella copri	11129	155265	7.168
Ruminococcus bromii	9564	155265	6.160
Alistipes putredinis	9238	155265	5.950
Collinsella aerofaciens	7903	155265	5.090
Escherichia coli	7854	155265	5.058
Parabacteroides distasonis	5051	155265	3.253
Subdoligranulum variabile	4561	155265	2.938
Bacteroides ovatus	4363	155265	2.810
Clostridium clostridioforme	3719	155265	2.395
Bacteroides eggerthii	3590	155265	2.312
[Eubacterium] biforme	3458	155265	2.227
Bacteroides caccae	2914	155265	1.877
Dorea longicatena	2767	155265	1.782
Roseburia faecis	2089	155265	1.345
Haemophilus parainfluenzae	1840	155265	1.185
Alistipes onderdonkii	1736	155265	1.118
Bacteroides coprophilus	1481	155265	0.954
Ruminococcus callidus	1423	155265	0.916
Lactobacillus mucosae	1285	155265	0.828
Bacteroides fragilis	1218	155265	0.784
Blautia obeum	1212	155265	0.781
Alistipes indistinctus	1033	155265	0.665
Coprococcus eutactus	861	155265	0.555
Alistipes finegoldii	829	155265	0.534
Barnesiella intestinihominis	798	155265	0.514
Collinsella stercoris	688	155265	0.443
Dorea formicigenerans	644	155265	0.415
[Ruminococcus] torques	632	155265	0.407
Clostridium butyricum	596	155265	0.384
Roseburia inulinivorans	588	155265	0.379
Lactobacillus salivarius	573	155265	0.369
Prevotella stercorea	549	155265	0.354
Veillonella dispar	466	155265	0.300

Eggerthella lenta	453	155265	0.292
Blautia producta	420	155265	0.271
Oxalobacter formigenes	286	155265	0.184
Butyrivibrio crossotus	278	155265	0.179
Streptococcus luteciae	263	155265	0.169
Bulleidia p-1630-c5	254	155265	0.164
[Eubacterium] cylindroides	213	155265	0.137
Desulfovibrio D168	212	155265	0.137
Clostridium ramosum	197	155265	0.127
Coprobacillus cateniformis	138	155265	0.089
Lactococcus garvieae	120	155265	0.077
[Ruminococcus] gnavus	113	155265	0.073
Clostridium spiroforme	92	155265	0.059
Streptococcus alactolyticus	84	155265	0.054
[Eubacterium] dolichum	73	155265	0.047
Streptococcus anginosus	65	155265	0.042
Coprococcus catus	64	155265	0.041
Lactobacillus iners	36	155265	0.023
Mitsuokella multacida	33	155265	0.021
Ruminococcus flavefaciens	31	155265	0.020
Streptococcus sobrinus	30	155265	0.019
Bifidobacterium adolescentis	21	155265	0.014
Malus x domestica	21	155265	0.014
Clostridium symbiosum	20	155265	0.013
Lactobacillus ruminis	20	155265	0.013
Rothia mucilaginosa	19	155265	0.012
Clostridium perfringens	18	155265	0.012
Lactonifactor longoviformis	16	155265	0.010
Clostridium bolteae	15	155265	0.010
Clostridium saccharogumia	15	155265	0.010
Denitromonas indolicum	14	155265	0.009
Streptococcus infantis	13	155265	0.008
Lactobacillus plantarum	8	155265	0.005
Clostridium lavalense	7	155265	0.005
Staphylococcus aureus	7	155265	0.005
Bifidobacterium bifidum	6	155265	0.004
Lactobacillus reuteri	6	155265	0.004
Lactobacillus vaginalis	5	155265	0.003
Leuconostoc mesenteroides	5	155265	0.003

Rothia dentocariosa	5	155265	0.003
Brachymonas denitrificans	3	155265	0.002
Aggregatibacter segnis	2	155265	0.001
Bifidobacterium longum	2	155265	0.001
Lactobacillus zeae	2	155265	0.001
Veillonella parvula	2	155265	0.001
Weissella viridescens	2	155265	0.001
